# Transcription Factor RFX2 Is a Key Regulator of Mouse Spermiogenesis

**DOI:** 10.1038/srep20435

**Published:** 2016-02-08

**Authors:** Yujian Wu, Xiangjing Hu, Zhen Li, Min Wang, Sisi Li, Xiuxia Wang, Xiwen Lin, Shangying Liao, Zhuqiang Zhang, Xue Feng, Si Wang, Xiuhong Cui, Yanling Wang, Fei Gao, Rex A. Hess, Chunsheng Han

**Affiliations:** 1 State Key Laboratory of Stem Cell and Reproductive Biology, Institute of Zoology, Chinese Academy of Sciences, Beijing, 100101, China; 2 Graduate University of Chinese Academy of Sciences, Beijing, 100049, China; 3 Comparative Biosciences, College of Veterinary Medicine, University of Illinois, Urbana, IL 61802-6199, USA

## Abstract

The regulatory factor X (RFX) family of transcription factors is crucial for ciliogenesis throughout evolution. In mice, *Rfx1-4* are highly expressed in the testis where flagellated sperm are produced, but the functions of these factors in spermatogenesis remain unknown. Here, we report the production and characterization of the *Rfx2* knockout mice. The male knockout mice were sterile due to the arrest of spermatogenesis at an early round spermatid step. The *Rfx2-*null round spermatids detached from the seminiferous tubules, forming large multinucleated giant cells that underwent apoptosis. In the mutants, formation of the flagellum was inhibited at its earliest stage. RNA-seq analysis identified a large number of cilia-related genes and testis-specific genes that were regulated by RFX2. Many of these genes were direct targets of RFX2, as revealed by chromatin immunoprecipitation-PCR assays. These findings indicate that RFX2 is a key regulator of the post-meiotic development of mouse spermatogenic cells.

Spermatogenesis is a complex yet highly regulated developmental process that involves the proliferation and differentiation of spermatogonia, the meiosis of spermatocytes and post-meiotic development of spermatids, which includes the formation of the acrosome, condensation of the chromatin, disposal of extra cytoplasm, and generation of the flagellum^1^. The post-meiotic development, also known as spermiogenesis, is dependent on the accurate expression of a large number of testis-specific genes, the disruption of which often results in spermatogenic defects and male infertility^2^,^3^. Up to now, a limited number of transcriptional factors have been reported to be directly involved in the regulation of these testis-specific genes^4^,^5^,^6^. Bioinformatics screening for tissue-specific regulatory motifs in mouse genes revealed that the transcription factor binding sites of regulatory factor X (RFX) were significantly enriched in the promoters of testis-specific genes^7^,^8^,^9^, indicating that RFX transcriptional factors may be potential regulators and important for mouse spermatogenesis.

RFX proteins were initially identified in mammals as the regulatory factor that binds to the X-box motif in MHCII gene promoter^10^. In vertebrates, this family consists of seven members (RFX1-7) characterized by a highly conserved 76-residue winged-helix DNA binding domain^11^, with similar DNA-binding specificities^12^. RFX proteins are critical for development and involved in various devastating disease conditions. Their most prominent function that has been conserved throughout evolution is the pivotal role in regulating ciliogenesis. Cilia, including flagella, are microtubule-based organelles that project from the surface of most eukaryotic cells and have evolved to perform diverse roles in motility, signaling, and sensory reception^13^,^14^,^15^. Defects in ciliary assembly and function result in various disorders commonly known as ciliopathies. In *C. elegans*, the RFX transcription factor DAF19 is expressed in ciliated sensory neurons and its disruption results in the absence of sensory cilia^16^. Two *Rfx* genes, *dRfx* and *dRfx2*, have been identified in *D. melanogaster*. The *dRfx* gene was essential for the differentiation of ciliated sensory neurons^17^. Knockdown of *Rfx2* in *Xenopus* interfered with cilia assembly and resulted in cilia-defective embryonic phenotypes^18^,^19^, while in zebrafish *Rfx2* knockdown resulted in reduced Kupffer’s Vesicle ciliary length and perturbations in left-right asymmetry^18^,^19^. In mice, RFX family members have diverse functions, with RFX3 and RFX4 linked to ciliogenesis. *Rfx3*-deficient mice displayed frequent left-right asymmetry defects caused by abnormalities in nodal cilia^20^. Analysis of *Rfx4* mutant (RFX4^L298P^) mice demonstrated that RFX4 modulates SHH signaling by regional control of ciliogenesis^21^. Although the RFX homologs broadly participated in regulating ciliogenesis throughout the evolutionary tree, none has been reported to be required for sperm flagellum biogenesis, which is intrinsically similar to ciliogenesis.

*Rfx1-3* have been reported to be highly expressed in mouse testis twenty years ago^12^, and *Rfx4* more recently^22^. Using mutant mice models, the functions of RFX1, RFX3 and RFX4 have been well investigated^20^,^21^,^23^. However, despite the high expression of these genes in mouse testis and their potential roles in regulating numerous testis-specific genes, none has been reported to play an essential role in mouse spermatogenesis. Up to now, the physiological functions of RFX2 in mouse development have remained obscure, but its potential role in spermatogenesis is particularly interesting for following reasons: 1) The expression of *Rfx2* in the testis is several hundred fold higher than in other tissues^12^. 2) *Rfx1-4* mRNAs are expressed at high levels in round spermatids, but only *Rfx2* is highly expressed from pachytene spermatocytes to round spermatids^24^,^25^. 3) Previous studies reported that RFX2 is a potential regulatory factor for several testis-specific genes, such as germline-specific *H1t* and *Alf*^24^,^26^.

To investigate the role of RFX2 in mouse spermatogenesis, we inactivated *Rfx2* using homologous recombination. We report that male *Rfx2*^*−/−*^ mice were sterile due to complete blockage of spermatogenesis in the round spermatid phase of spermiogenesis. Round spermatids did not generate flagella and failed to differentiate into elongated spermatids, becoming detached from the seminiferous epithelium either individually or in clusters. Disruption of spermiogenesis was accompanied by apoptosis, associated with altered mRNA levels of a large number of genes that are involved in multiple processes of spermatogenesis. These data reveal a novel function for the *Rfx* gene family in mammalian spermatogenesis and demonstrate that *Rfx2* is essential for the completion of round spermatid differentiation and flagellar biogenesis.

## Results

### Targeted Inactivation of Mouse *Rfx2* Disrupts Spermatogenesis

**Figure 1:**
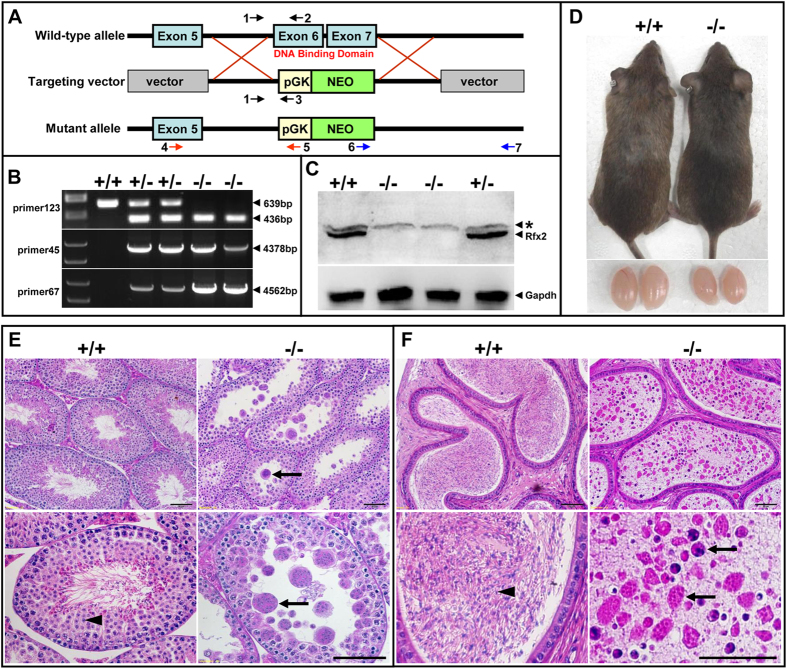
Spermatogenesis in *Rfx2*^*−/−*^ mice is arrested in the round spermatid phase. (**A**–**D**) Targeted disruption of the *Rfx2* gene. (**A**) Schematic representation of the wild-type allele, the targeting vector and the mutated allele. *Rfx2* gene was inactivated by replacing exon 6 and 7 with a phosphoglycerate kinase promoter-neomycin-resistance gene cassette. Arrows and numbers indicate primers used in PCR genotyping. (**B**) The gene knockout was confirmed by PCR genotyping. The genomic DNA isolated from the mouse tails was amplified with primer pairs specific for the WT (primers 1 and 2: 639 bp) and mutant (primers 1 and 3: 436 bp; primers 4 and 5: 4378 bp; primers 6 and 7: 4562 bp) *Rfx2* alleles. (**C**) Western blot analysis showing the absence of RFX2 protein in total testis extracts of *Rfx*2^*−/−*^ mice. *indicates a non-specific band recognized by anti-RFX2 antibody. (**D**) Comparison of the body and testis sizes of adult WT (+/+) and *Rfx2* deficient (−/−) mice. (**E**,**F**) Spermatogenesis in *Rfx2*^*−/−*^ mice was blocked in the round spermatid phase. (**E**) Histological analysis of sections from *Rfx2*^*−/−*^ and WT testis. Arrowhead indicates normal round spermatids, arrows indicate multinucleate giant cells. (**F**) Histological analysis of epididymis from WT and mutant mice. Arrowhead indicates mature sperm while arrows indicate degenerating multinucleate giant cells. Scale bar, 50 μm.

To investigate the function of RFX2 *in vivo*, we disrupted the *Rfx2* gene in mouse embryonic stem cells by replacing exon 6 and 7 with a phosphoglycerate kinase–neomycin-resistance cassette through conventional gene targeting (Fig. 1A). Exon 6 and 7 were chosen for targeting because they encode the DNA binding domain of RFX2^11^,^12^. Deletion of these two exons also resulted in a pre-mature termination of protein translation due to a shift of the reading frame. One correctly targeted embryonic stem cell clone was used to generate chimeric mice that transmitted the mutated allele through the germline. Heterozygous mice were crossed to generate *Rfx2*^*−/−*^ mice. Genotyping of the mice was performed by PCR using specific primers recognizing either the wild-type (WT) or mutant alleles (Fig. 1B). RT-PCR experiments revealed that the deletion of exon 6 and 7 results in the splicing of exon 5 to exon 8, which leads to a frame shift and premature termination at an out-of-frame stop codon. Western blot analysis showed that RFX2 protein was absent in the testicular extracts of the *Rfx2*^*−/−*^ mice (Fig. 1C). Thus, the deletion of exon 6 and 7 constitutes a strong loss-of-function mutation.

Of a total of 92 mice born from 9 independent F1 x F1 crosses, 20 *Rfx2*^*−/−*^ mice were derived, indicating normal Mendelian transmission with no significant embryonic lethality. No differences in body size and weight between WT and *Rfx2*^*−/−*^ mice were observed shortly after birth. However, approximately 25% of the *Rfx2*^*−/−*^ mice showed severe growth retardation with age (Supplementary Fig. S1), and these animals died before 2 months of age. Most of the surviving mutant mice grew normally and showed no obvious external abnormalities or anatomical aberrations (Fig. 1D); however, some of the survived mutant mice showed slight growth retardation. *Rfx2*-deficient mice with normal growth were chosen for further investigation. Female *Rfx2*^*−/−*^ mice were fertile, and no histological abnormality in the ovaries was observed (Supplementary Fig. S2). However, no pregnancy was established when male *Rfx2*^*−/−*^ mice were mated to WT females despite the formation of copulatory plugs. Testes from adult *Rfx2*^*−/−*^ males on average were 30% smaller by weight than those from WT males. Histological examination of *Rfx2*^*−/−*^ testes revealed that spermatogenesis was arrested at an early round spermatid step. Round spermatids failed to undergo morphological development into elongating spermatids and became detached from the seminiferous epithelium, either individually or in clusters as multinucleate giant cells (Fig. 1E). Epididymal tubules from adult WT mice were filled with sperm, whereas those from *Rfx2*^*−/−*^ mice contained large numbers of degenerating round spermatids and multinucleate giant cells (Fig. 1F). The complete absence of sperm in the epididymis of the *Rfx2*^*−/−*^ mice explains why *Rfx2*^*−/−*^ mice were infertile despite normal sexual behavior.

### Spermatogenesis is Normal up to the Round Spermatid Phase in Rfx2^*−/−*^ Mice

**Figure 2:**
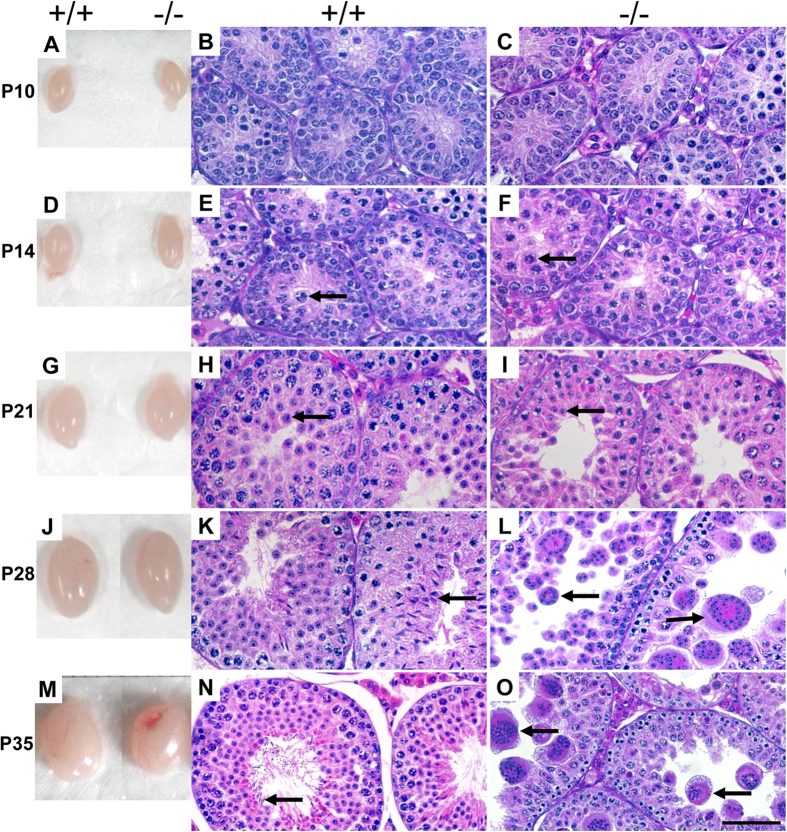
RFX2 is required during the first-wave of spermatogenesis. Sizes and histology of testes from WT (+/+) and mutant (−/−) mice at various ages were evaluated visually or by HE staining. No significant difference in size was detected between WT and mutant testes until postnatal day 21 (P21); however, mutant testes are smaller than WTs since P28. Arrows indicate early pachytene spermatocytes (**E,F**), round spermatids (**H,I**), elongated spermatids (**K**), spermatozoa (**N**) and multinucleate giant cells (**L,O**). Scale bar, 50 μm.

To identify the earliest stage at which defective spermatogenesis occurred, testes from *Rfx2*^*+/+*^ and *Rfx2*^*−/−*^ mice at different ages were isolated and compared. At P10 and P14, testes of both WT and RFX2 mutant mice were similar in size and histological analysis demonstrated no obvious differences in seminiferous tubular size and the population of germ cell types (Fig. 2A–F). At P21, round spermatids were present in both WT and *Rfx2*^*−/−*^ mice (Fig. 2H,I). Notably, spermatogenesis was delayed at this phase in some mutant animals. There were fewer tubules containing round spermatids and when present, the round spermatids were fewer in number than in the WT mice (Supplementary Fig. S3). Starting from P28, testes from *Rfx2*^*−/−*^ mice were significantly smaller than WT (Fig. 2J,M). At P28, elongating spermatids were abundant in WT mice but completely absent in the mutant testes, with numerous detached round spermatids in almost every seminiferous tubular lumen (Fig. 2K,L). At P35, the first wave of spermatogenesis was completed and mature spermatozoa were present in WT testis (Fig. 2N). However, no spermatozoa were detected in the *Rfx2*^*−/−*^ testes and degenerating multinucleate giant cells with dark staining nuclei were frequently observed (Fig. 2O), similar to the defects seen in the adult mutant mice.

**Figure 3:**
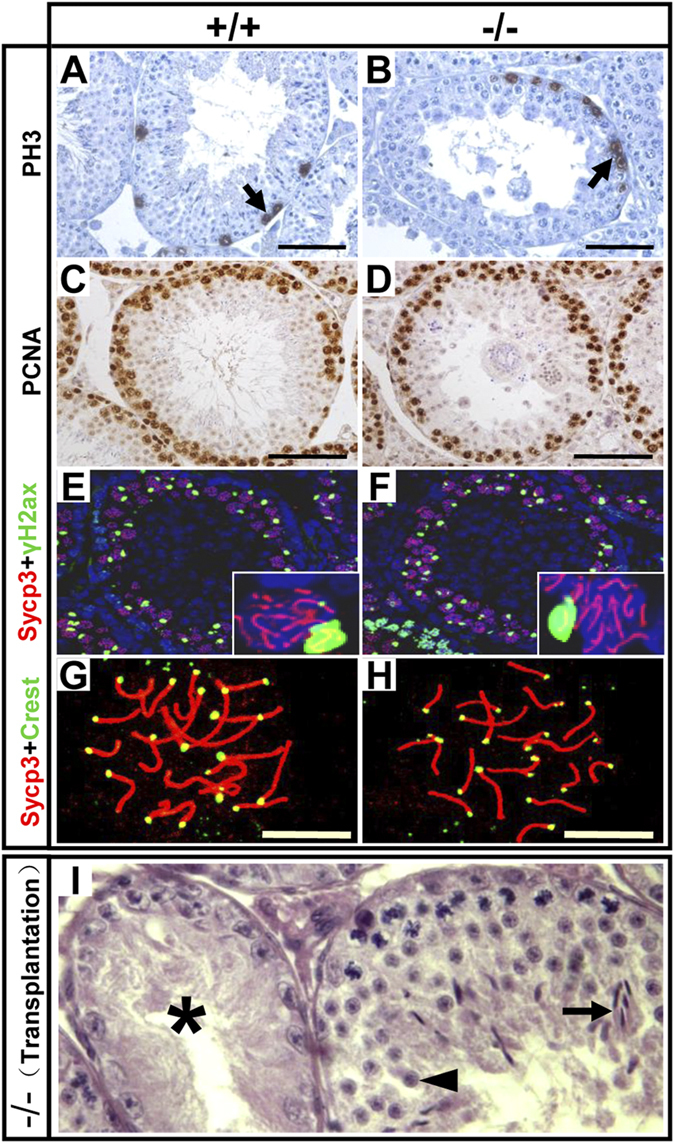
Loss of *Rfx2* does not interfere with the mitotic and meiotic phases of spermatogenesis. (**A**–**D**) Loss of *Rfx2* did not impair germ cell proliferation. Sections of WT and *Rfx2* deficient testes were immunostained with anti-PH3 (**A**,**B**) and anti-PCNA (**C**,**D**) antibodies, respectively. Arrows indicate spermatogonia. Scale bar, 50 μm. (**E**–**H**) Meiosis was normal in *Rfx2* deficient mice. (**E**,**F**) Sections of WT and *Rfx2* deficient testes were immunostained with the anti-SYCP3 and anti-γH2AX antibodies. (**G**,**H**) Chromosome spreads of spermatocyte were immunostained for SYCP3 and CREST. Scale bar, 10 μm. (**I**) Reconstituted spermatogenesis in busulfan-pretreated *Rfx2* mutant testis by transplanted WT spermatogonia. The asterisk (*) represents a non-colonized tubule that lacks germ cells. The right tubule has been colonized by WT spermatogonia. Arrowhead and arrow indicate normal round and elongated spermatids, respectively.

To determine more specifically the cause of spermatogenic arrest in the round spermatid phase in mutant testes, we examined the expression of recognized marker genes associated with the regulation of spermatogenesis. Immunostaining for WT1 and PLZF showed that the numbers of Sertoli cells and undifferentiated spermatogonia, respectively, were comparable between WT and *Rfx2*^*−/−*^ mice (Supplementary Fig. S4). Also, no significant difference was observed between the two groups in immunostaining for PCNA and PH3 (Fig. 3A–D), markers for proliferating cells and M-phase spermatogonia, respectively, in seminiferous tubules^27^, which indicated that normal germ cell proliferation occurred in the absence of RFX2. Next, the progression of meiosis was analyzed, using immunostaining for SYCP3, phospho-H2AX and CREST. Phospho-H2AX marks DNA double strand breaks before the synaptonemal complex is fully established and the unsynapsed X and Y chromosomes, while SYCP3 and CREST mark the synaptonemal complex and centromeres, respectively. Homologous chromosome synapsis and sex body formation were indistinguishable between *Rfx2*^*−/−*^ and WT mice (Fig. 3E–H and Supplementary S4). Together, these data suggest that the mitotic and meiotic phases of spermatogenesis in *Rfx2*^*−/−*^ mice were normal and that germ cell failure was inherent to the post meiotic phase of spermatogenesis.

Spermatogenic failure can result from defects either in germ cells themselves or in other aspects including somatic environment and hormonal regulation^28^. Since we inactivated *Rfx2* using a conventional knockout strategy, this issue was addressed by using the spermatogonial stem cell transplantation assay. WT spermatogonia were transplanted into seminiferous tubules of the *Rfx2*^*−/−*^ mice, which were pretreated with busulfan to eliminate endogenous germ cells. As shown in Fig. 3I, spermatogenesis was successfully reconstituted in a subset of the tubules of the recipient mice, with successful completion of both round spermatid differentiation and the production of elongated spermatids. These studies demonstrate that spermatogenic failure observed in *Rfx2* mutant mice is a germ cell autonomous defect.

### Early Arrest of Spermiogenesis and Germ Cell Apoptosis in Rfx2 mutant Mice

**Figure 4:**
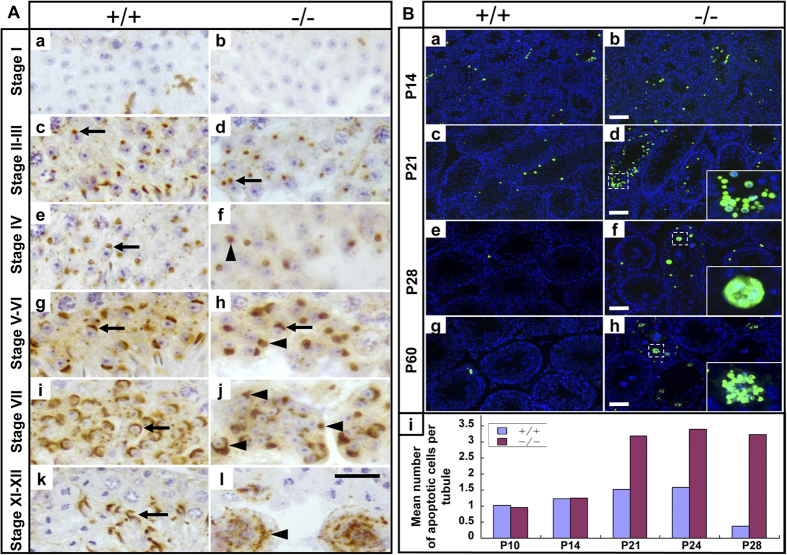
Impaired spermiogenesis and germ cell apoptosis in *Rfx2* mutant mice. (**A**) Spermiogenesis was arrested at an early step in *Rfx2* mutant mice. Testis sections of adult WT and mutant mice were immunostained for the acrosome marker AFAF. Arrows indicate normal acrosomes of different steps as indicated. Arrowheads show abnormal acrosomes in mutant mice. Scale bar, 25 μm. (**B**) Absence of *Rfx2* resulted in germ cell apoptosis. TUNEL staining of testis sections from WT (a,c,e,g) and mutant (b,d,f,h) mice at P14 (a,b), P21 (c,d), P28 (e,f) and P60 (g,h). Insets (f,g) show apoptotic multinucleate giant cells. (i) Quantitative results of apoptotic cells detected in sections from WT and mutant mice. Scale bar, 50 μm.

Mouse spermiogenesis is subdivided into 16 spermatid steps based on the shape of the developing acrosome of the spermatids^29^,^30^. We performed immunostaining of the acrosomal protein AFAF^31^ to determine the precise step at which spermiogenesis was arrested in *Rfx2*^*−/−*^ testes. As shown in Fig. 4A, AFAF first appeared as a large round vesicular shaped structure adjacent to the nucleus of steps 2–3 round spermatids in both WT and mutant tubules (Fig. 4A-c, A-d). By stage IV, WT round spermatids had acrosomes that were beginning to flatten over the nucleus, while in mutants, many spermatids had large round acrosomes or acrosomic vesicles that were not flattened (Fig. 4A-e, A-f). By stage V-VI, a few mutant spermatids had developed small arc-shaped acrosomes as in WT mice, but most spermatids had irregular-shaped acrosomes (Fig. 4A-g,A-h). By stage VII, WT spermatids had large arc-shaped acrosomes that covered the nuclei, but mutants had abnormal acrosomes of varying shapes (Fig. 4A-i,A-j). The spermatids with abnormal acrosomes degenerated (Fig. 4A-i), and no spermatids beyond step 7 were observed. These data show that most *Rfx2*^*−/−*^ round spermatids did not mature beyond step 7 of spermiogenesis.

Multinucleate giant cells, similar to those observed in the *Rfx2* mutant mice, have been previously shown to contain apoptotic elements^4^. Therefore, the TUNEL assay was performed to determine whether loss of RFX2 causes germ cells apoptosis. In WT testes, apoptotic germ cells were readily observed at P14 and P21 and then their number dropped to a level barely detectable at P28 and P60 (Fig. 4B). In *Rfx2*^*−/−*^ testes, apoptosis was similar to WT mice at P14, but significantly higher at all subsequent postnatal days. The number of apoptotic cells in mutant mice was more than twice that seen in WT mice at P21 and 8-fold higher than in WT mice at P28, when apoptotic multinucleate giant cells were observed in *Rfx2*^*−/−*^ testes (Fig. 4B-i). The results indicate that loss of *Rfx2* inhibits germ cell differentiation and promotes germ cell apoptosis, at the time when acrosomal formation also begins to fail.

### RFX2 Is Required for Sperm Flagellum Assembly

**Figure 5:**
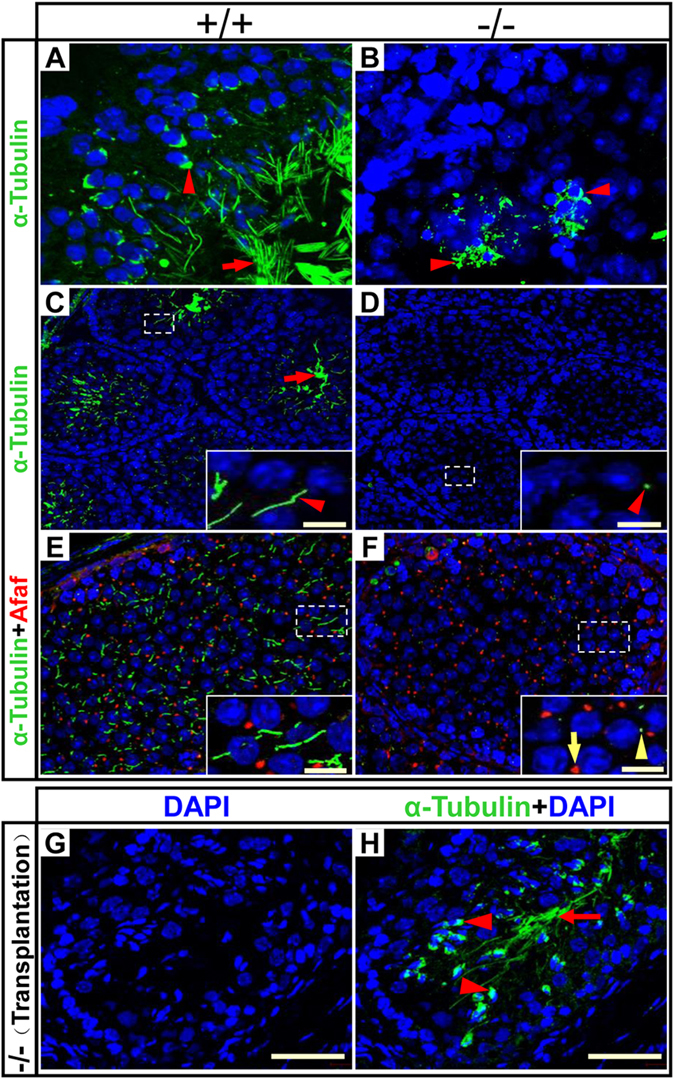
*Rfx2* mutants have defects in sperm flagella assembly. (**A**–**D**) Testis sections of adult mice (**A,B**) or P24 mice (**C,D**) were stained with antibody against **α**-tubulin (green). **α**-tubulin was located in the manchette (arrowhead) and flagella (arrow) of normal spermatids, while in mutant mice, the manchette structure (arrowhead) of degenerating spermatids was still stained with **α**-tubulin but the flagella were undetectable. Scale bar, 10 μm. (**E,F**) AFAF (red) and **α**-tubulin (green) staining of P24 mice testis sections. Round spermatids at an early step in mutant mice had normal acrosome (arrow) while the flagella were missing. Only small green dots representing unassembled flagella were observed (arrowhead). Scale bar, 10 μm. (**G,H**) Sections from *Rfx2* mutant testis transplanted with WT germ cells were stained for **α**-tubulin. Arrowheads indicate manchette structure and the arrow indicates flagellum. The nuclei were stained with DAPI. Scale bar, 50 μm.

RFX2 has been reported to regulate ciliogenesis of vertebrates^18^. The sperm flagellum is a special type of motile cilium, with a typical “9+2” microtubule arrangement^15^,^32^. In the adult WT testis, flagellar microtubules in round spermatids, elongating spermatids and mature sperm, as well as those supporting the manchette structure in elongating spermatids, were readily revealed by the α-tubulin immunostaining (Fig. 5A). In contrast, typical flagellar microtubules could not be detected in adult *Rfx2*^*−/−*^ mice. Instead, a degenerative manchette structure was observed in multinucleate giant cells detached from the tubule wall. At P24, round spermatids were abundant in both WT and mutant mice, with normal morphology, and strong immunofluorescence of α-tubulin was detected in tubules of WT mice, but only a few dot-shaped signals were seen in the mutant seminiferous tubules (Fig. 5C,D). Double immunofluorescence staining for AFAF and α-tubulin revealed in WT mice that round spermatids developed a round acrosomal vesicle and a typical short flagellum in steps 2–3 of spermiogenesis (Fig. 5E). In mutant mice, these early steps of round spermatids had a similar round acrosomal vesicle (AFAF^+^), as in the WT; however, flagellum assembly was blocked and only dot-like α-tubulin signal were observed (Fig. 5F). When WT spermatogonia were transplanted into the testes of mutant mice, the manchette and flagella of elongating spermatids were observed, similar to that of WT mice (Fig. 5H). These results demonstrate that RFX2 is required for flagellum assembly during spermiogenesis.

### RFX2 Does Not Regulate the Expression of Known Regulators of Spermatogenesis

**Figure 6:**
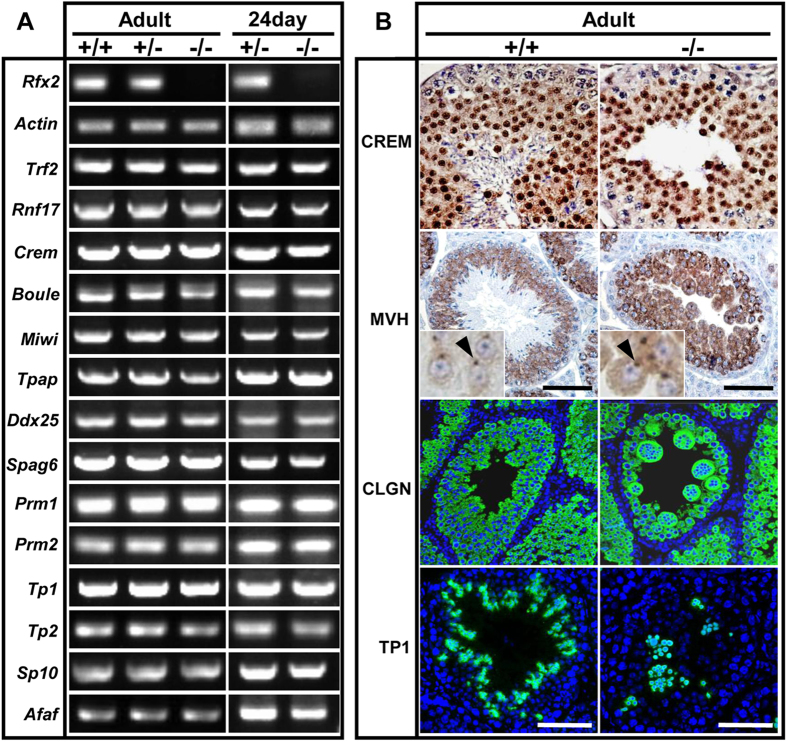
RFX2 does not act through known spermatogenesis regulators. (**A**) RT–PCR analysis of gene expression. The expression of transcription factors *Trf2* and *Crem* as well as other important spermiogenesis genes were not affected by Rfx2 knockout. *Actin* was used as the internal control. (**B**) Sections of WT and *Rfx2* deficient testes were immunostained with the anti-CREM, anti-MVH, anti-CLGN and anti-TNP1 antibodies, respectively. The expression pattern of these proteins between (+/+) and (^*−/−*^) were indistinguishable. Arrowheads indicate chromatoid bodies labelled by MVH. Scale bar, 50 μm.

Loss of *Rfx2* induced spermatogenesis arrest in the round spermatid phase. This phenotype is similar to several previously reported KO mice in which genes such as *Crem, Trf2, Rnf17, Miwi, Boule, Tpap* and *Ddx25* were inactivated^3^,^4^,^5^,^33^,^34^,^35^,^36^,^37^. In *Rfx2*^*−/−*^ testes, the mRNA levels of these genes were comparable to those expressed in WT mice (Fig. 6A and Supplementary Fig. S5). Also, no difference was found between WT and mutants in the expression of a number of important postmeiotic genes, including Transitional Protein 1/2 (*TP1/2*), and Protamine 1/2(*Prm1/2*) and in the acrosomal maker genes *Sp10* and *Afaf*, as well as a potential RFX2 target gene *Spag6* 26(Fig. 6A). Immunostaining for CREM, MVH, CLGN and TP1 was also present in round spermatids of the mutant mice, including detached multinucleate giant cells, similar to those in WT mice (Fig. 6B). Immunostaining for MVH revealed that chromatoid bodies were present in the round spermatids of *Rfx2* mutant mice and TP1 was detected in the detached degenerating spermatids of the mutant mice but also in WT elongating spermatids (Fig. 6B). These results indicate that RFX2 acts either through different pathways or downstream of these key regulators of spermatogenesis.

### RFX2 Is a Key Regulator of Cilia/Flagella-related Genes and some Testis-specific Genes

**Figure 7:**
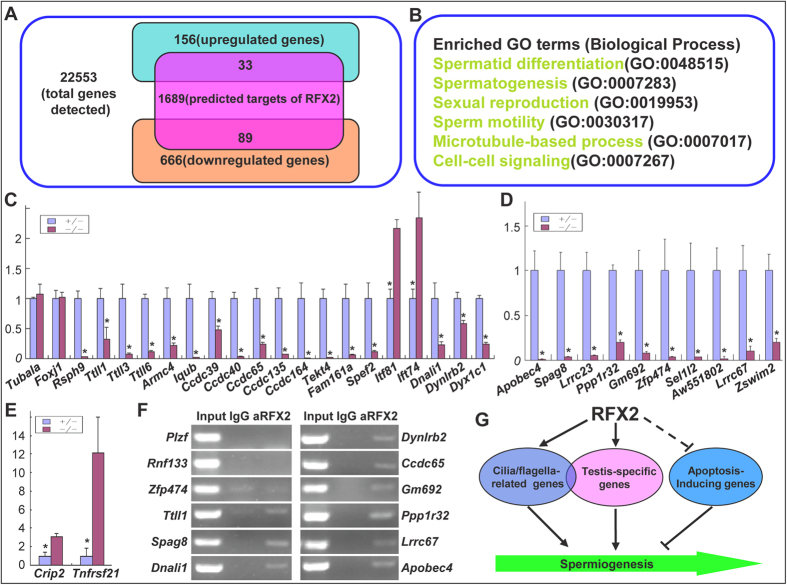
Analysis of RFX2-dependent gene expressions during mouse spermatogenesis. (**A**) Numbers of differentially expressed genes in heterozygous and *Rfx2* mutant testis and predicted target genes of RFX2. (**B**) Gene Ontology terms significantly enriched among differentially expressed genes. (Biological process category only; P value < 0.05). (**C**–**E**) Real-time PCR validation of cilia/flagella-related genes (**C**), testis-specific genes (**D**) and apoptosis-inducing genes (**E**) selected from the differentially expressed genes. Data presented as mean ± SD were obtained from at least three individual testes. *p < 0.01. (**F**) ChIP-PCR validation of some predicted RFX2 target genes in (**C**,**D**). Two negative controls were included using primers flanking genomic segments of *Plzf* and *Rnf133* genes that were predicted not to contain binding sites of RFX2. (**G**) A model on the involvement of RFX2 in the regulation of spermatogenesis. Solid lines indicate direct transcriptional control and dashed line indicates undefined regulatory mechanism.

To identify RFX2-dependent genes during spermatogenesis, we carried out RNA-seq analysis using P24 heterozygous and mutant testes samples. We chose P24 samples because spermiogenesis in mutants was normal before this time point, but thereafter massive detachment of the round spermatids was observed. RNAs were isolated from the total testicular cells, and two independent biological samples were prepared for each genotype. The reads from the biological duplicates were pooled for further analysis after confirming the correlation coefficient on mRNAs were higher than 0.9. Based on the p-values provided by the Cuffdiff program (cutoff value p < 0.05), 156 up-regulated and 666 down-regulated genes were identified among a total of 22,553 genes detected (Fig. 7A and Supplementary Fig. S6). Scanning the proximal promoters (from -1000 bp to 1000 bp of the transcription start sites) of these genes, using our home-developed software, revealed that 122 genes were potential target genes of RFX2 as they contained at least one typical RFX-binding site^38^ (Fig. 7A).

Functional annotation of these 822 differentially expressed genes using the DAVID web tool^39^ indicated that the up-regulated genes were not enriched with key testicular terms, while the down-regulated genes were enriched with terms such as “sperm motility, spermatogenesis, spermatid differentiation, microtubule-based process” (Fig. 7B and Supplementary Fig. S7), consistent with RFX2 being an important regulator of spermiogenesis and flagellum assembly (described above). As we were interested in the cilia/flagella-related and testis-specific genes, an intensive literature search was made to identify those involved in cilia/flagella assembly, structure, or function. These included the following: tubulin tyrosine ligase-like 1/3/6 (*Ttll1/3/6*), dyslexia susceptibility 1 candidate 1 (*Dyx1c1*), dynein light chain roadblock-type 2 (*Dynlrb2*), light intermediate chain (*Dnali1*), intraflagellar transport 74/81 (*Ift74/81*), sperm flagellar 2 (*Spef2*), family with sequence similarity 161, member A (*Fam161a*), *Tektin4*, radial spoke head 9 (*Rsph9*), armadillo repeat containing 4 (*Armc4*), IQ motif and ubiquitin domain containing (*Iqub*), five members of coiled-coil domain-containing family (*Ccdc39/40/65/135/164*) and others. Most of the genes were significantly downregulated except for *Ift74* and *Ift81*, which were upregulated, and for *Tubala* and *Foxj1*, which were not changed in the mutant mice (Fig. 7C). All the real-time PCR results correlated well with the RNA seq data (Supplementary Fig. S8).

Of the 822 differentially expressed genes, 140 genes were predicted or previously reported to be highly or specifically expressed in testis^40^, of which 3 genes were up-regulated and 137 genes were down-regulated (Supplementary Fig. S6). We chose 10 testis-specific genes for further validation by real-time PCR quantification, using *Gapdh* as an internal control. Again, all these genes were significantly down-regulated in the mutant mice (Fig. 7D). In contrast, the expressions of two apoptosis-inducing genes, *Crip2* and *Tnfrsf21*^41^,^42^, were up-regulated (Fig. 7E). Nine of the 19 verified cilia/flagella-related genes and all 10 verified testis-specific genes were predicted to be RFX2 target genes (Supplementary Fig. S9). ChIP-PCR was performed using antibodies against RFX2 to examine these predictions. Using promoter specific primers, 9 of the 10 promoters examined were immunoprecipitated by the RFX2 antibody, in comparison with isotype IgG (Fig. 7F). These results demonstrated that RFX2 is a key regulator of cilia/flagella-related genes and numerous testis-specific genes during mouse spermatogenesis.

## Discussion

This study demonstrates that the transcription factor RFX2 plays an essential role in spermatogenesis during post-meiotic development of the spermatids. Loss of *Rfx2* gives rise to two discrete defects in spermiogenesis: a) the arrest of spermiogenesis in an early round spermatid step accompanied by apoptosis, and b) the failure to assembly the flagellum. Analysis of RFX2-dependent gene expressions resulted in the identification of a large number of cilia/flagella-related genes and testis-specific genes that are regulated by RFX2 (Fig. 7G).

RFX proteins have long been known to be critical for ciliogenesis in animals from worms to vertebrates. Here, we report for the first time that RFX2, a highly conserved member, plays an essential role in sperm flagellum assembly by regulating many cilia/flagella-related genes. These genes encode proteins that are involved in diverse aspects of cilia/flagella assembly and function, including components of the axoneme (*Ccdc39, Ccdc40, Ccdc65, Ccdc164, Dnali1, Dyx1c1* and *Rsph9*), the ciliary basal body (*Fam161a*), ciliary tubulin posttranslational modification (*Ttll1, Ttll3* and *Ttll6*), intraflagellar transport (*Ift74* and *Ift81*), and accessory structures of the flagellum (*Tekt4, Spef2*). Many of these genes have been shown previously to be essential for the formation of the flagellum. For example, the deletions of *Spef2* and *Ttll1* resulted in a truncated or absent flagellum^43^,^44^. In mice, RFX2, 3 and 4 are involved in ciliogenesis, and loss of either *Rfx3* or *Rfx4* resulted in stunted cilia but not their loss^20^,^21^. In contrast, assembly of the flagellum in *Rfx2* mutant mice was severely disrupted, resulting in the loss of all sperm flagella. Therefore, RFX2 may act upstream of the other regulators in flagellar biogenesis and other family members cannot compensate for its role.

Primary ciliary dyskinesia (PCD) is a genetically heterogeneous autosomal recessive disorder characterized by dysfunction of the respiratory cilia and sperm flagella, as well as laterality defects^45^. Inactivation of many of the RFX2-regulated cilia-related genes has been shown to result in PCD. These genes include *Ccdc39, Ccdc40, Ccdc65, Ccdc164, Rsph9, Ttll1, Spef2* and *Dyx1c1*^43^,^46^,^47^,^48^,^49^. Bioinformatics analysis showed that most of these genes were potential target genes of RFX2. *Ccdc65* and *Ttll1* were chosen for further verification by ChIP assay and both genes were proven to be targets of RFX2. Considering that some *Rfx2*-deficent mice showed severe growth retardation and previous studies showed that RFX2 is essential for the development of left–right asymmetry in Xenopus^18^,^19^, we speculated that RFX2 may also participate in ciliogenesis in other systems. We generated KO mice of mixed genetic background of 129/s6 and C57BL/6. The 129/s6 ES cells were used to generate the chimeric male mice, which were crossed to C57BL/6 females to generate the F1 heterozygotes. Therefore, genetic background variation in F2 homozygotes may result in mice with defects only in spermatogenesis as well as mice with defect(s) in other system(s) displayed as smaller body size. Although we did not observe disruption of the left-right asymmetry in the surviving KO mice, it is possible that other RFX-family members were able to compensate for the loss of RFX2 function. Alternatively, as a result of evolution, RFX2 may serve a specialized role promoting ciliogenesis in testicular germ cells. Further investigation of RFX2 will help to better understand the molecular mechanisms of ciliogenesis and contribute to the development of diagnostic and therapeutic applications in the treatment of PCD.

It is well recognized that numerous testis-specific genes are expressed during spermatogenesis, especially in round spermatid steps^50^,^51^. However, the mechanisms for transcriptional regulation of these genes are largely unknown. Several testis-specific genes have been shown to be potential direct targets of RFX2^24^,^52^,^53^ and the current study revealed a large number of these genes to be regulated by RFX2. Among the cilia/flagella-related genes regulated by RFX2, only three (Armc4, Iqub, Tekt4) are specifically expressed in the testis. Therefore, the other testis-specific genes regulated by RFX2 represent additional classes of proteins that could regulate other cellular events, which when disrupted in the KO mice could contribute to the other abnormalities associated with spermatogenic arrest, such as detachment of round spermatids from Sertoli cell adhesions and the formation of multinucleated giant cells, as well as apoptosis. Loss of some additional RFX2-regulated genes may not result in observable histopathological abnormalities due to the premature detachment of round spermatids and increase in cellular death.

Up to now, a limited number of genes (*Crem, Trf2, Rnf17, Miwi, Boule, Tpap* and *Ddx25*) have been identified as key regulators of mouse spermiogenesis, and they share similar phenotypes with *Rfx2*^3^,^4^,^5^,^33^,^34^,^35^,^36^,^37^. Except for three transcription factors (CREM, TRF2, RFX2), the rest are related to RNA post-transcriptional processing, storage, and/or translation regulation. One prominent phenotype of all these gene knockouts, including *Rfx2*, is that spermiogenesis is arrested at a round spermatid step, with round spermatids sloughing from the tubule in the form of multinucleate giant cells accompanied with massive apoptosis. This severe abnormality of spermiogenesis reflects that these key regulators control the expression of a large number of genes from diverse families. The other common feature is that meiosis, in most cases, appears normal, although these regulators begin their expressions in the spermatocytes. Likely, such regulators are expressed at an earlier stage in order to activate or suppress the expression of their target genes at transcription or the post-transcriptional level, while the protein targets are needed post-meiotically. The third shared feature is that these key genes do not appear to regulate each other, but rather to define distinct pathways of spermiogenesis. However, these regulators could share common upstream activators, as is the case for *Rfx2* and *Miwi,* which are potential direct targets of A-MYB, a master regulator of male meiosis^26^,^54^.

In conclusion, the results presented in the present study demonstrate that RFX2 is a key regulator of sperm flagellum assembly and other aspects of round spermatid differentiation by regulating numerous genes during mouse spermiogenesis. This work expands our understanding of the functions of the mammalian RFX family transcription factors in ciliogenesis and in mammalian spermatogenesis.

## Materials and Methods

### Gene Targeting and Mice

The targeting construct was based on PL253^55^. A 9.9 Kbp genomic fragment containing the middle region of *Rfx2* was subcloned into PL253. The *Rfx2* gene was disrupted by replacing exon 6 and 7, which encode the DNA binding domain, with the neomycin-resistance cassette by using homologous recombination. The resulting targeting vector was linearized with Not1 and electroporated into 129/s6 embryonic stem (ES) cells. ES clones were selected with G418 and ganciclovir, and G418-resistanct clones were screened by long PCR using a primer internal to NEO gene and a primer upstream to the 5′ end or downstream to the 3′ end of the gene targeting region. One ES clone of correct targeting was injected into C57BL/6 blastocysts, and male chimeric mice were backcrossed with C57BL/6 mice to generate heterozygous KO mice. Genotyping of subsequent mice were conducted by PCRs of genomic DNA isolated from tail tips using the following primers: primer 1, 5′- TCCACCTCTAGCCAACTCT-3′; primer 2, 5′- TCCTGTCTTGGGTCTATCCT -3′ and primer 3, 5′- ATGTGGAATGTGTGCGAG -3′ (Fig. 1). The primer 1 and primer 2 generated a 639 bp DNA fragment that identified the WT allele, while the primer 1 and primer 3 primers yielded the 436 bp fragment of the targeted allele. Mice used in these studies had a mixed genetic background of 129/s6 and C57BL/6. Experimental protocols were approved by the Animal Care and Use Committee of the Institute of Zoology, Chinese Academy of Science, and all experiments were performed in accordance with the approved guidelines.

### Histology, Immunohistochemistry and Immunofluorescence

Mouse testes were fixed in Bouin’s solution (Sigma-Aldrich, MO, USA) overnight at 4 °C. Paraffin embedding, section preparation, deparaffinization, H&E staining or immunohistochemistry analysis were carried out by using standard histological procedures. For Immunofluorescence, testes were embedded in OCT compound (Sakura Finetek) and frozen in liquid nitrogen. Cryosections (5 μm) were mounted on glass slides and subjected to immunostaining. Primary antibodies were incubated at 4 °C overnight and secondary antibodies were added at room temperature for 2 hr. Nuclei were visualized by staining with DAPI. The following antibodies were used: rabbit polyclonal anti-SYCP3 (ab-15093, Abcam), mouse monoclonal anti-γH2AX Ser-139 (cat. no. 05–636, Millipore), rabbit polyclonal anti-CLGN (12629-1-AP, Protein Tech Group), CREST serum (Immunovision, Springdale, AR), mouse monoclonal anti-α-tubulin (Sigma), rabbit polyclonal anti-MVH (ab13840, Abcam), mouse monoclonal anti-DAZL (LS-C188293, LifeSpan Biosciences), rabbit polyclonal anti-WT1 (Epitomics), mouse anti-PLZF (Calbiochem), rabbit anti-PH3 (Cell Signaling Technology), rabbit anti-CREM (X-12, Santa Cruz), rabbit polyclonal anti-AFAF (a kind gift from Prof. Yi-Xun Liu, Institute of Zoology, Beijing)^31^

### Western Blot Analysis

Western blot analysis was performed as previously described^56^. Anti-RFX2 antibody (C-15, Santa Cruz, CA, USA) and anti-GAPDH (FL-335, Santa Cruz, CA, USA) were used to detect RFX2 and GAPDH respectively.

### Spermatocyte Nuclei Spreads

Spermatocyte nuclear spreads were prepared as described^57^.

### TUNEL Assay

TUNEL assay was performed with the Deadend Fluorometric TUNEL system (Promega) according to the manufacturer’s protocol.

### Chromatin Immunoprecipitation (ChIP)

ChIP assays were performed using a mixture of enriched germ cells and a kit (Upstate Cell Signaling Solutions, NY, USA) according to manufacturer’s instructions with slight modifications. Briefly, mixed enriched germ cells were isolated from the testes of WT adult mice by two-step enzymatic digestion. In the first step, albuginea-removed testes were digested with 1 mg/ml type IV collogenase (Sigma) and 500 μg/ml DNase I (Sigma) for 5 min at 37 °C to get the seminiferous tubules. In the second step, the tubules were digested with 0.25% trypsin (Invitrogen) and 500 μg/ml DNase I at 37 °C for 5 min into single cells, and then the cells were collected and fixed with 1% formaldehyde in PBS for 10 minutes at 37  °C to crosslink DNA and protein. Many somatic cells, especially those between and outside of the seminiferous tubules are removed by the first step of enzymatic digestions, and the cells harvested in the second step digestion were enriched with germ cells. The cells were then washed with iced-cold phosphate buffered saline (PBS) and lysed with 1 ml lysis buffer [1% SDS, 10 mM EDTA, 50 mM Tris, pH 8.1 1 mM PMSF, protease inhibitor cocktail (Sigma)]. Lysates were incubated on ice for 10 minutes and sonicated on ice with an ultrasonic sonicator (Omni-Ruptor 250) at 20% power and 40% pulse for 7 min. The resulting DNA fragments were between 0.2 to 1 kb in length. The sonicated cell lysates was centrifuged at 13,000 rpm for 10 minutes at 4 °C to remove cell debris. 200ul supernatant were diluted 10-fold in dilution buffer (0.01% SDS, 1.1% Triton X-100, 1.2mM EDTA, 16.7mM Tris-HCl, pH 8.1, 167mM NaCl) and then incubated with 5 micrograms of anti-RFX2 antibody (C-15, Santa Cruz, CA, USA) or normal goat IgG as a negative control at 4 °C overnight. The immuno complexes were collected with 50ul ProteinA/G Sepharose beads at 4 °C for 3 hours and precipitated at 2000 rpm at 4 °C for 2 minutes. The precipitated beads were washed orderly with each immunoprecipitation buffers. Following immunoprecipitation, RFX2-bound DNA fragments were eluted and subjected to PCR reaction. ChIP-PCR primers are listed in Supplementary Table S2, and all the primers were designed to be centered over the predicted RFX binding sites of the promoters.

### Germ Cell Transplants

Germ cell transplants were carried out as previously described^58^.

### PCR and RNA sequencing

Total RNA was extracted from 24-day-old or adult mouse testes by using Trizol according to the standard protocol (Invitrogen) as previously described^56^. To perform RT-PCR amplification, reaction mixtures were first denaturalized at 94 °C for 3 min, 30 cycles with the following conditions were then carried out: 30 s of denaturalization at 94 °C, 30 s of annealing at 58 °C, 45 s of extension at 72 °C. Subsequently, the reaction was incubated at 72 °C for 10 min. The PCR products were verified by agarose gel electrophoresis. qPCRs were conducted with UltraSYBR Mixture (Beijing CoWin Biotech, Catalogue Number CW0956) by following the manufacturer’s instructions on a LightCycler 480 platform (Roche). The primer pairs of selected genes were listed in Supplementary Table S1. Many primers were obtained from PrimerBank^59^. For RNA sequencing, total RNA was extracted from testes of the 24-day-old heterozygous or mutant mice. DNA contaminants were eliminated by RNase-free DNase treatment. The quality of the RNA samples was analyzed by agarose gel electrophoresis and by RT-PCR detection the expressions of selected genes. Then the RNA samples were subjected to RNA sequencing following manufacturer’s recommendations (Novogene) using Illumina Hiseq 2000 instrument. Two biological replicates were included for each genotype. 53 million reads and 62 million reads were generated for the two samples of KO mice, and 63 million reads and 59 million reads were generated for the two control mice respectively. Data analysis was performed as previously described^60^. Briefly, RNA-seq reads generated from each sample were aligned to the mouse genome (UCSC mm9) with Tophat-2.0.6. Mapped reads were subsequently assembled into transcripts guided by reference annotation (mm9, USCS gene annotation) with Cufflink-2.0.2. Differential gene and expression analysis was conducted by using the Cuffdiff program. The number of Fragments Per Kilobase per Million (FPKM) is used by Cuffdiff to represent the expression level of a gene. This means that the read numbers of genes were normalized by the gene lengths and the sequencing depths. The fold change value of a gene between KO and WT mice is the ratio of its two FPKM values. The sequencing data have been submitted into the NCBI GEO database (accession number GSE74961).

### Prediction of RFX2 binding sites

We developed a program to scan the proximal promoter regions of interested genes for transcription factor binding sites (TFBSs). Detailed description of the algorithm will be published in a separate paper. Briefly, the promoter sequences of a gene from humans and mice 10 Kbp upstream and 5 Kbp downstream of the transcription start sites were aligned to retrieve the conserved regions, which are about 2 Kbp long altogether and for which the sequence identity is above 80%. The positional weight matrix (PWM) of RFX2 was used to scan for its binding sites. The second exons of all the protein coding genes, which were about 1.5 Mbp long, were used as the negative control set as they seldom contain TFBSs. The match score cutoff value was chosen so that on average no more than 3 TFBSs were identified in 10-Kbp sequences of the negative control set.

## Additional Information

**How to cite this article**: Wu, Y. *et al.* Transcription Factor RFX2 Is a Key Regulator of Mouse Spermiogenesis. *Sci. Rep.* **6**, 20435; doi: 10.1038/srep20435 (2016).

## Supplementary Material

Supplementary Information
