# Supplementary material for: Transcription Factor RFX2 Is a Key Regulator of Mouse Spermiogenesis
Source: Sci Rep. 2016 Feb 8;6:20435. doi: 10.1038/srep20435 (PMC4745085; doi:10.1038/srep20435)
Supplement: Supplementary Information [file srep20435-s1.doc]

# Transcription Factor RFX2 Is a Key Regulator of Mouse Spermiogenesis

Yujian Wu^1^, Xiangjing Hu^1^, Zhen Li^1,2^, Min Wang^1^, Sisi Li^1,2^, Xiuxia Wang^1^, Xiwen Lin^1^,Shangying Liao^1^, Zhuqiang Zhang^1^，Xue Feng^1^，Si Wang^1,2^, Xiuhong Cui^1^，Yanling Wang^1^, Fei Gao^1^，Rex A. Hess^3^, Chunsheng Han^1*^

^1^ State Key Laboratory of Reproductive Biology, Institute of Zoology, Chinese Academy of Sciences, Beijing, 100101, China

^2^ Graduate University of Chinese Academy of Sciences, Beijing, 100049, China

^3^ Comparative Biosciences, College of Veterinary Medicine, University of Illinois, Urbana, IL 61802-6199, USA

* Correspondence: [hancs@ioz.ac.cn](mailto:hancs@ioz.ac.cn) Fax: +86-10-64807105

**Supplemental Materials**

**Supplemental Data:**

**Figure S1. Some mutant mice showed severe growth retardation with age.** Comparison of the body size of control (+/+) and *Rfx2* deficient (-/-) mice at P10, P15 and P20, respectively.

**Figure S2. Female *Rfx2^-/-^* mice are fertile.** (A) Fertility of *Rfx2*-null Male and Female. Males and females of the three different genotypes were tested for fertility with wild-type males and females. Data presented as mean ± SD. (B) Histological analysis of ovaries from wild-type and mutant mice showed no obvious abnormalities in the *Rfx2^-/-^* ovaries. Bar, 100 µm.

**Figure S3. Spermatogenesis was delayed in some mutant mice at P21.** In some mutant mice, the numbers of tubules containing round spermatids and of round spermatids in these tubules were significantly less than the control mice. The asterisks represent tubules containing round spermatids, and arrows indicate round spermatids. Bar, 50 µm.

**Figure S4. Normal WT1 and PLZF staining and normal chromosome pairing in *Rfx2^-/-^* testis.** (A-D) Testis sections from control and mutant mice were stained for WT1 and PLZF. The numbers of sertoli cell (A, B) and undifferentiated spermatogonia (C, D) are comparable between control and *Rfx2^-/-^* mice. (E) Immunofluorescence images of chromosome spreads stained with anti-SYCP3 and anti-γH2ax antibodies. Meiotic chromosome pairing and sister chromatid (SC) formation is normal in *Rfx2^-/-^* mice.

**Figure S5.** **The expression of known spermatogenesis regulators are not affected in *Rfx2* mutant mice**. Real-time PCR validation of the known regulators of spermatogenesis. Data presented as mean ± SD were obtained from at least three individual testes. *p < 0.01.

**Figure S6. Fold change of differentially expressed genes in *Rfx2* mutant testis.** (A) Fold change of all differentially expressed genes in Rfx2 mutant testis. (B) Fold change of differentially expressed genes which are highly or specifically expressed in testis.

**Figure S7. Gene Ontology terms significantly enriched among down-regulated genes in *Rfx2* mutant testis.** (A) Gene Ontology terms significantly enriched among down-regulated genes in *Rfx2* mutant testis. (B) Gene Ontology terms significantly enriched among down-regulated genes which are highly or specifically expressed in testis. (Biological process category only; P value <0.05)

**Figure S8. Plots of the mRNA expression level of selected genes based on the RNA-seq FPKM values.**

**Figure S9. X box motifs present in the 5' control region of many downregulated cilia/flagella-related genes and testis-specific genes**. The X-box consensus sequence that is simplified from the standard consensus motif (5’-GTNRCC(0-3N)RGYAAC-3’ ) is shown at the top, where N is any nucleotide, R is a purine and Y is a pyrimidine . A gap aligned with the two NNs just means that the hit has one N. The location of these motifs is given relative to the transcriptional start sites.

| **Table S1. qPCR primers.** | |  |  |
| --- | --- | --- | --- |
| **Gene** | **Forward primer 5' to 3'** | | **Reverse primer 5' to 3'** |
| *Ift74* | GAGGAAACGAAGAATCAGGAACT | | TTCAGCTCTTGGTTGGTGATG |
| *Ift81* | TTCATCGTGGACAGCCTCAAT | | CATCTTGCTTGGGGTCAATCT |
| *Dynlrb2* | GAATCCAGAGTCACAAAGGGG | | GACCCGCATACTGAACCGTT |
| *Ttll1* | GAAGTGGGTCACTGACATTGAG | | ACGTTGCGAATGGTTTGCAC |
| *Ttll3* | AGAGAAGTCACAGTCCATCTCC | | AGCCTCATCGACAAAGTCTGA |
| *Ttll6* | AAGCCCTTCATCATCGACGG | | TGTCTAGGTTAGGGTGGGAGTAA |
| *ccdc39* | GAGCGAAGAACATTTTAAGGCCA | | TTCAAGTCATCCAGCTTTTGAGT |
| *Ccdc40* | GCCCTGAGGTATCACCACAG | | CCCCCGCCTTCAGTTGTAATG |
| *Ccdc65* | CCCCTGTCCGAGGAAGATCA | | GAGGATGGTTCTCCACTGCG |
| *Ccdc135* | AGGAGGCTGAGCGTGAAGA | | AGTTGGTGGTATAGGAAGGAGG |
| *Ccdc164* | GAACTGTGGGAGATGCTCAAC | | TGATGTCTTCCGACTGTTTCTTC |
| *Spef2* | ACCGTGAGTCCCAAGTCATTT | | GTTGAACCCCTGCTCTCTGAA |
| *Rsph9* | CACTGCTCACGTCCCTTATGC | | GCGATGTAGTAATCCGCCACA |
| *Iqub* | ATGTCTGATCCCGAGGAAGAA | | CTCTGGCATCACATCTGACCC |
| *Fam161a* | AAAGCTAAATCCTTGACACCCAA | | TGGCTCGGAATTTCTTCTTACAC |
| *Tekt4* | GGCCCCACAGTCAATAGATGT | | GGGCATAAGAGTTCTGAAACCA |
| *Armc4* | GGACTGCTGCGGGATATGAG | | AATGCTGAGGGTTCCAAATATGT |
| *Wdr69* | AATTGCCACGGGGTCTTTTGA | | TGAATGTCCCATAATTTGGCTGT |
| *Dnali1* | GGTGAGCCGGAATACAGAGAA | | TGTTTGGTAGGATCTGGGACA |
| *Dyx1c1* | TGCGCGATGCTGACGTATT | | AGAAAGGCTATCCCACAGAACT |
| *Foxj1* | CCCTGACGACGTGGACTATG | | GCCGACAGAGTGATCTTGGT |
| *Tuba1a* | CGCCCAACCTACACTAACCTA | | CCAGAGGGAAGTGGATACGAG |
| *Tnfrsf21* | GCCATGTTGACCGTACCACT | | CAGACTCGCAGGCTCATGTT |
| *Crip2* | CAAGGACTGGCACAAGTTCTG | | CCCCGATGTTCACGCCTTT |
| *Gm692* | ATGCCGACCCTGGGAGTAAA | | AGCCTCTGCTTAGTCAATGCC |
| *Lrrc23* | AGATGATGTGGATGACGTTGATG | | CAGAGTAATGAGAGCCCTTCCT |
| *Apobec4* | GGAGTCACAACCACCCAGAAG | | AGCCTCGTTACAAGGGGAGTT |
| *Sel1l2* | GTGTCTGTGAACTAGGCCACT | | CATATCCCATTTCGGCAAGCA |
| *Spag8* | CCTGGACAGGGTCCTAAGTTC | | GGAGGTTTATATGGCCCTCTCA |
| *Zswim2* | CTTCGCGGAGGCTGTAAAG | | GCAGGAGATAGATGCTGCTACTC |
| *Zfp474* | CACAAGGAACTATCCACCTCTCC | | GTTTTTGGTTAGACCGCCACA |
| *Ppp1r32* | CTATGGTCGGGAGGAGTTTAAGC | | TAGGGGAGAGTAGCTCTGACT |
| *Lrrc67* | CTGACGGTGGATTTAATTGCCA | | AGAGAGAGGTCATCAATCGAGTC |

| **Table S2. CHIP-PCR primers.** | |  |  |
| --- | --- | --- | --- |
| **Gene** | **Forward primer 5' to 3'** | | **Reverse primer 5' to 3'** |
| *Rnf133* | AGCTCTTGCTGAAACATGC | | GACTTGAATAGCCAACCCA |
| *Lrrc67* | TAATCTGCCTGGGCGTTTG | | GGCTTCTCACCGCTCTGTC |
| *Gm692* | CCCTAATCCCTGCACCTTA | | CCTCATACTTTCTGCCGTTT |
| *Ppp1r32* | CTGTTGCTAGGTGCTGGTT | | ACCTCCCTGTGGAAGTTGA |
| *Apobec4* | AAGTGTCAGAGCCGTATCCT | | GCACCTGTGCCATCAAGAC |
| *Spag8* | GGAAGACACGAACACATCAAG | | GTCTCCTGGAGTTGTTTGC |
| *Dnali1* | TCTTCTCCCAACCTCCATC | | AACCGAAACAGACCTAACG |
| *Ttll1* | ATTACAGCAGTTGAAGGCACA | | ATACCGAGGCAGGAAAGAA |
| *Ccdc65* | CTGTTCTACGAAATCCGATGC | | CTCTGTCCTCCACGCCAAG |
| *Dynlrb2* | GGCTACCTACACTTGCCTGAC | | CTCCCAGATTCATCTCAACAC |
| *Plzf* | GGCTCCGTAAGCGGTAAGT | | CTAGCACCACCCGCCTAAA |
